# Hereditary alpha-1-antitrypsin deficiency and its clinical consequences

**DOI:** 10.1186/1750-1172-3-16

**Published:** 2008-06-19

**Authors:** Laura Fregonese, Jan Stolk

**Affiliations:** 1 Alpha1 International Registry (AIR), c/o Department of Pulmology, Leiden University Medical Center, Leiden, The Netherlands

## Abstract

Alpha-1-antitrypsin deficiency (AATD) is a genetic disorder that manifests as pulmonary emphysema, liver cirrhosis and, rarely, as the skin disease panniculitis, and is characterized by low serum levels of AAT, the main protease inhibitor (PI) in human serum. The prevalence in Western Europe and in the USA is estimated at approximately 1 in 2,500 and 1 : 5,000 newborns, and is highly dependent on the Scandinavian descent within the population. The most common deficiency alleles in North Europe are PI Z and PI S, and the majority of individuals with severe AATD are PI type ZZ. The clinical manifestations may widely vary between patients, ranging from asymptomatic in some to fatal liver or lung disease in others. Type ZZ and SZ AATD are risk factors for the development of respiratory symptoms (dyspnoea, coughing), early onset emphysema, and airflow obstruction early in adult life. Environmental factors such as cigarette smoking, and dust exposure are additional risk factors and have been linked to an accelerated progression of this condition. Type ZZ AATD may also lead to the development of acute or chronic liver disease in childhood or adulthood: prolonged jaundice after birth with conjugated hyperbilirubinemia and abnormal liver enzymes are characteristic clinical signs. Cirrhotic liver failure may occur around age 50. In very rare cases, necrotizing panniculitis and secondary vasculitis may occur. AATD is caused by mutations in the *SERPINA1 *gene encoding AAT, and is inherited as an autosomal recessive trait. The diagnosis can be established by detection of low serum levels of AAT and isoelectric focusing. Differential diagnoses should exclude bleeding disorders or jaundice, viral infection, hemochromatosis, Wilson's disease and autoimmune hepatitis. For treatment of lung disease, intravenous alpha-1-antitrypsin augmentation therapy, annual flu vaccination and a pneumococcal vaccine every 5 years are recommended. Relief of breathlessness may be obtained with long-acting bronchodilators and inhaled corticosteroids. The end-stage liver and lung disease can be treated by organ transplantation. In AATD patients with cirrhosis, prognosis is generally grave.

## Disease name and synonyms

Alpha-1-antitrypsin deficiency

Alpha1-protease inhibitor deficiency

## Definition

Alpha-1-antitrypsin deficiency is a genetic disorder that manifests clinically as pulmonary emphysema, liver cirrhosis and much less frequently as the skin disease panniculitis.

## Background

Alpha-1-antitrypsin deficiency was first reported in 1963 by Carl-Bertil Laurell and Sten Eriksson who noted a link between low plasma serum levels of alpha-1-antitrypsin and symptoms of pulmonary emphysema [1]. Since these first cases were described, an understanding of the biochemical mechanisms and genetic abnormalities involved has developed [2], and alpha-1-antitrypsin deficiency is now thought to be one of the most common hereditary disorders worldwide, comparable in frequency to cystic fibrosis [3,4].

**Figure 1:**
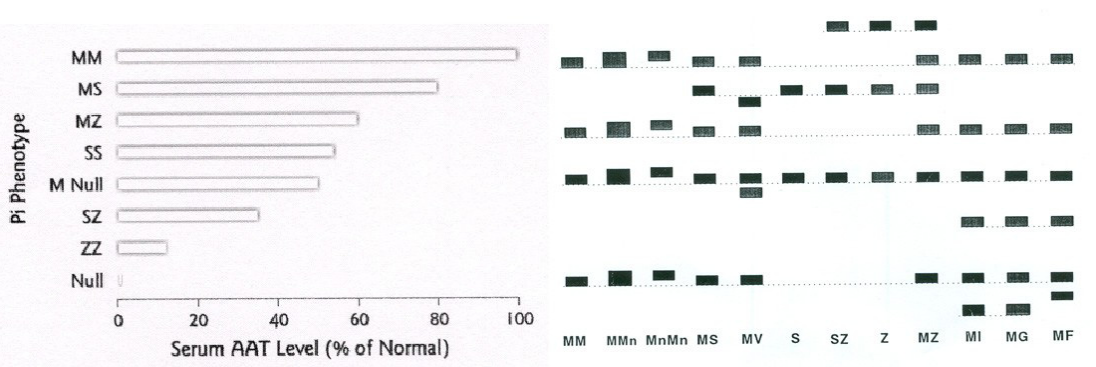
**Serum alpha-1-antitrypsin (AAT) levels in various Pi phenotypes consisting of two alleles each, *i.e*. MM (left part of the figure)**. Serum iso-electric focusing (Pi) is one of the methods to determine the type of allele deficiency present in patients (right part of the figure). Each Pi type gives a characteristic set of bands from top to bottom on a polyacramide gel and is reported as Pi phenotype. These Pi types correlate to mean general serum AAT concentrations, but are dependent on the inflammatory status of the individual, as AAT is an acute-phase reactant protein. Clinical significant liver disease only occurs with Pi ZZ, clinical significant lung disease is associated with Pi Null, Pi SZ and PiZZ types.

The protein alpha-1-antitrypsin is a 52 kDa molecule produced primarily in hepatocytes and released into the blood circulation by the liver [5]. The protein is present in all body tissues but appears to have its primary physiologic significance in the lungs, where it protects the healthy but fragile alveolar tissue from proteolytic damage by enzymes like neutrophil elastase [6]. Alpha-1-antitrypsin is an acute phase protein which means that production by the liver is subject to various stimuli, including inflammatory mediators induced by fever [2]. Therefore, protein levels in the circulation may vary depending on the medical condition of an individual. The normal serum concentration may range between 1.5 to 3.5 g/L (or 20 to 48 **μ**M) [2]. Among clinicians and patients some confusion may arise about the clinical significance of deficiency in alpha-1-antitrypsin. The first cases described by Laurell and Eriksson were related to a very low serum concentration of the protein and subsequently it was discovered that these patients had a particular gene defect (defined as Z deficiency) in both alleles of the chromosome making them prone to the development of lung and liver disease. Later in time it was discovered that various other gene defects exist and most of them cause their own range of serum concentrations below the normal range [5]. A serum concentration below 0.5 g/L (11 **μ**M) is considered a reason for further analysis. In addition, individuals who carry a genetic defect in only one allele of the chromosome (heterozygotes) may also have a reduced serum concentration (summary in Figure 1). Often, when patients with emphysema are told that they are deficient of alpha-1-antitrypsin, they fear that their deficiency will cause the clinical events described by Laurell and Eriksson. However, as described below, it will be explained why in contrast to heterozygous MZ type, in most cases only the homozygous Z type of deficiency, as well as some very rare Null phenotypes with absent alpha-1-antitrypsin serum levels result in clinical significant disease.

## Epidemiology

The low frequency of the Pi ZZ phenotype in the general normal population makes firm data collection with respect to prevalence of affected individuals difficult to obtain. The prevalence of AAT deficiency in newborns has been estimated from large population studies, with a screening of all newborns in Sweden in 1972 to 1974 being most comprehensive [7]. Of 200,000 children in that study, 127 had the PiZZ phenotype, yielding a prevalence rate of approximately 1 in 1,600 newborns. Studies from various regions of Europe have shown a large variation in frequency of the Z gene in different countries [8]. The gene frequency is highest on the northwestern seaboard of the European continent and the mutation seems likely to have arisen in southern Scandinavia. In the USA, therefore, Z gene frequencies are highest in individuals of northern of western European descent [9]. Over all, the prevalence in the general population in Western Europe is approximately 1 in 2,500. The distribution of the S gene is quite different: the gene frequency is highest in the Iberian Peninsula and the mutation is likely to have arisen in that region [8].

## Clinical description

The clinical manifestations of AAT deficiency are strongly associated with the ZZ type of deficiency, but vary widely between patients with ZZ type may, ranging from asymptomatic in some to fatal liver or lung disease in others [10]. In rare cases, necrotizing panniculitis and secondary vasculitis may occur, but the latter observation is presented in only one study and has not been reproduced in a second larger cohort.

### Liver disease and alpha-1-antitrypsin deficiency

**Figure 2:**
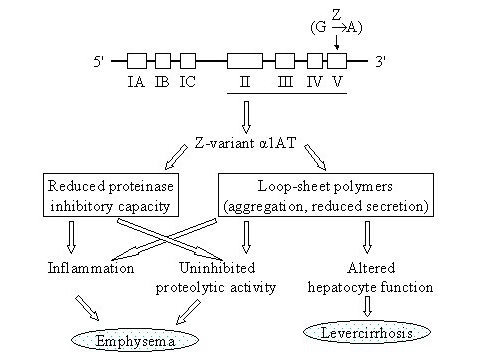
The Z-allele is the most important genetic defect in alpha-1-antitrypsin deficiency. It is a single mutation in exon 5 of the gene, leading to substitution of the amino acid glutamine (G) in position 342 in the protein for a lysine (A) amino acid. Alpha-1-antitrypsin is an important protease inhibitor, in particular of neutrophil elastase. The Z allele results in hepatic polymerization in both hepatocyte inclusions and decreased serum concentration. Therefore, strategies to augment the inherited deficiency as well as the development of small peptides that can selectively inhibit polymerization of the Z allele of the AAT protein in the liver are central to therapeutic approach.

Neonatal hepatic syndrome is a condition that occurs in a small percentage of newborns with homozygous Z genotype alpha-1-antitrypsin [7] (Figure 2). Prolonged jaundice after birth with conjugated hyperbilirubinemia and abnormal liver enzymes are characteristic clinical signs of the genotype. Bleeding problems may result from liver failure. This syndrome is believed to be an acute response to polymerization of the Z-type protein within hepatocytes [11,12].

The consequences of chronic polymerization of Z-type alpha-1-antitrypsin are hepatocyte damage and *via *fibrosis, a cirrhotic response [13]. This may occur clinically at childhood or more frequently at age above 50 [14]. The cirrhotic condition can be reversed by a liver transplant of a donor with M-type genotype alpha-1-antitrypsin [15]. In addition to cirrhotic disorder, a relatively high prevalence of hepatocellular carcinoma and cholangiocellular carcinoma has been reported [16].

### Lung disease and alpha-1-antitrypsin deficiency

The lung manifestations of AAT deficiency include emphysema and chronic obstructive pulmonary disease (COPD) [10]. Emphysema usually develops by the third to fourth decade in affected individuals who smoke cigarettes and may appear in the fifth or sixth decade in individuals who have never smoked.

It is currently hypothesized that two mechanisms contribute to the development of lung disease, in particular to emphysema (Figure 2). First, the serum level of alpha-1-antitrypsin appears to be important. It is observed that cigarette smokers who produce no alpha-1-antitrypsin at all in their liver or in monocytes, defined as individuals with the very rare homozygous Null variant, develop emphysema at younger age than subjects with the homozygous Z allele related deficiency [17]. Under appropriate circumstances, cigarette smoke recruits inflammatory cells in the lung which may release proteolytic enzymes in alveolar areas. For the development of emphysema, plasma levels of alpha-1-antitrypsin are important in the protection against proteolytic damage of alveoli in the lung, in particular by neutrophil elastase activity [18,19] (Figure 2). An issue further supported by the observation that individuals with SZ and SS genotype (although significantly less clinical information is available about SS type patients), who on average have higher levels of alpha-1-antitrypsin than the Null and Z genotype, are less prone to the development of emphysema [20].

Second, the Z mutant of alpha-1-antitrypsin has a point mutation Glu342Lys in the molecule that renders it prone to polymerization of the protein (polymers) [12] (Figure 2). These polymers co-localize with neutrophils in the alveoli of individuals with Z alpha-1-antitrypsin-related emphysema and animal experiments demonstrated that polymers cause an influx of neutrophils when instilled into murine lungs [21]. Such polymers exert their effect directly on neutrophils rather than *via *inflammatory cytokines. The transition of native Z alpha-1-antitrypsin to polymers inactivates its anti-proteinase function, and also converts it to a proinflammatory stimulus, further contributing to accelerated enzymatic alveolar tissue destruction.

**Figure 3:**
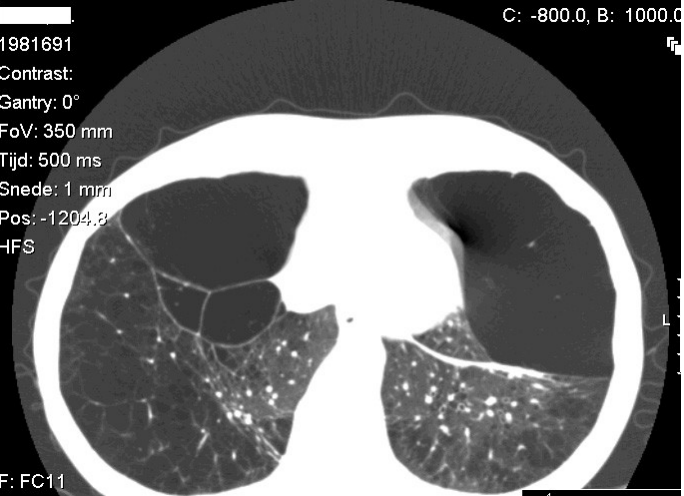
**Computed tomography of the lung with thin slices (1 mm) showing emphysema and bullae in the lower lung lobes of a subject with type ZZ alpha-1-antitrypsin deficiency**. There is also increased lung density in areas with compression of lung tissue by the bullae.

Textbooks on pulmonary medicine all teach us that cigarette smokers with the Z genotype around age 40 acquire panlobular emphysema in the lower lung lobes (Figure 3) with high probability. Yet, the importance of registries with phenotypical information about individuals with alpha-1-antitrypsin deficiency was recently illustrated [22]. The Alpha1 International Registry (AIR) database contains patients over age 40 with a smoking history of more than 10 packyears and Z genotype who have a completely normal lung function, including normal forced expiratory volume in the first second (FEV1) and normal gas transfer [22]. This suggests that protective factors may exist, an issue that is currently under intense investigation.

**Figure 4:**
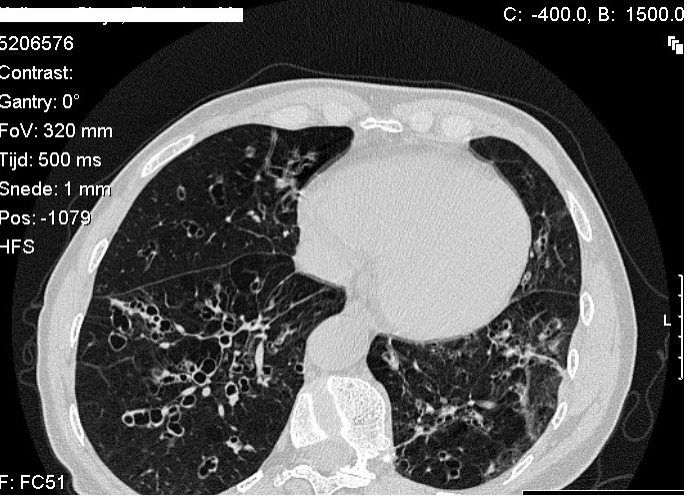
**Computed tomography of the lung with thin slices (1 mm) showing bronchiectasis in the lower lung lobes of a subject with type ZZ alpha-1-antitrypsin deficiency**. There are no signs of emphysema.

It is difficult to prove that other lung diseases, such as bronchiectasis (Figure 4), chronic bronchitis and asthma are associated with the homozygous Z genotype. This requires population based studies and these have not been conducted.

### Skin disorders and alpha-1-antitrypsin deficiency

It is generally assumed that the skin disorder panniculitis is associated with the Z genotype of alpha-1-antitrypsin [23]. Panniculitis manifests as spontaneous necrotic areas of the skin and spontaneous suppuration without previous trauma to the skin. It may occur anywhere in the body, but there is a preference to the gluteal areas, trunk, limbs and arms. Histologically, septae and fatty lobules are extensively altered by inflammation with lymphohistiocytic infiltration. Secondary vasculitis may results into necrosis which is eliminated transepidermally.

## Etiology

### Molecular biology of alpha-1-antitrypsin

The gene encoding alpha-1-antitrypsin is found on chromosome 14 at q31-32.3 and is called the *SERPINA1 *gene [24]. The gene comprises four coding exons and three additional exons. Hepatocytes and monocytes have promotors and they are distinct and work *via *different mechanisms. Promoters regulate basal expression in the steady state, while elements in the DNA known as enhancers modulate expression during inflammation [25]. Several sites of local transcriptional control have been identified upstream of the hepatocyte transcription initiation site, including binding sites for hepatocyte nuclear factor-1**α **(HNF-1**α**) and HNF-4. Humoral regulation is mediated by cytokines, particularly interleukin-6 (IL-6) and oncostatin-M [26]. These cytokines work *via *a 3' enhancer downstream of the hepatocyte promoter, and both oncostatin-M and IL-6 work *via *different response elements. Therefore, there are discrete binding sites providing distinct pathways for regulating the acute phase response via different cytokines [24].

Alpha-1-antitrypsin is also produced by monocytes and lung epithelial cells, and therefore there is also production present in the lung [5]. The promoter for the AAT gene in monocytes and other cell types is regulated by a different group of transcription factors known as the Sp1 family [27]. Here it has been suggested that RNA stability also influences expression, as some transcripts may be more stable than others. In the liver, there is only one transcript of AAT; however, in other cell types, there are a number of alternative transcripts. Although most serum AAT is manufactured in the liver, monocytes and lung tissue have the ability to make AAT; with appropriate stimuli, expression can be increased substantially [25]. Recently, McNab *et al*. showed that the defective gene in human monocytes of type Z alpha-1-antitrypsin deficient individuals can be corrected by transfection of small DNA fragments of M type alpha-1-antitrypsin to result in increased secretion of the protein [28]. The development of this method to repair *ex vivo *the gene defect in isolated human monocytes should have beneficial effects when these cells are re-infused in the circulation and retained in the lung to give protection against proteolytic alveolar damage. This experiment remains to be conducted.

Many hereditary variants of the *SERPINA1 *gene are known [29]. Because both alleles (paternal and maternal) can be detected, the hereditary mode is called codominant. The most common and clinically most important alleles leading to alpha-1-antitrypsin deficiency are the Z and S allele [24,30]. The frequency of the Z allele is highest in Scandinavian countries [6,29]; the frequency of the S allele is highest in Spain and Portugal [7,31].

### Protein characteristics of alpha-1-antitrypsin

**Figure 5:**
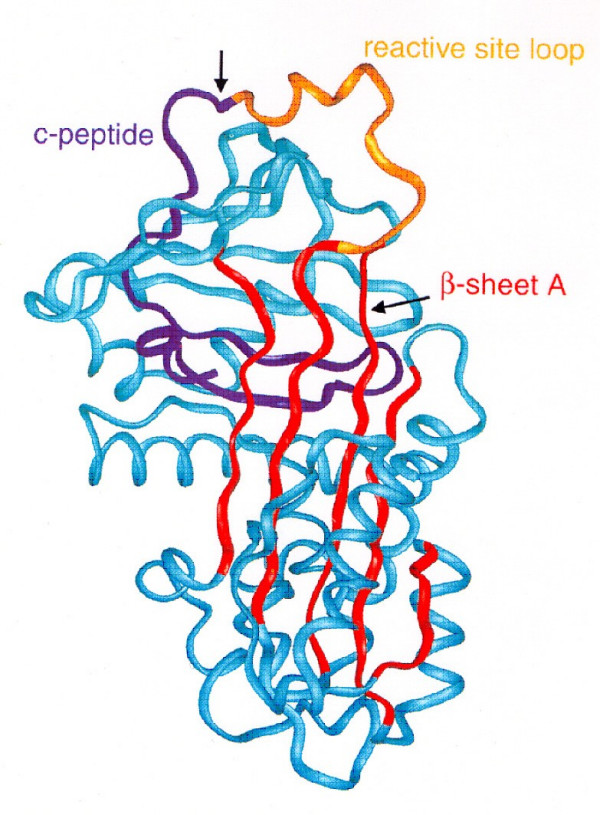
**Structure of alpha-1-antitrypsin: 3 **β**-sheets and 8 **α**-helixes**. The reactive loop site contains the neutrophil elastase binding site with a methionine residue. Reprinted from Janciauskiene S. Conformational properties of serine proteinase inhibitors (serpins) confer multiple pathophysiological roles. *Biochim Biophys Acta*, 2001, 1535:221-35. Copyright ^© ^2001, with permission from Elsevier.

The alpha-1-antitrypsin molecule includes 394 amino acids and 3 glycosylated side chains coupled to asparagine [32]. The molecule has a globular tertiary structure, and its active site to inhibit enzymes is on a surface protrusion (Figure 5). Here is where the most important amino acid is present, a methionine in position 358, an aminoacid susceptible to conversion to methionine sulfoxide by oxidants from cigarette smoke, rendering it much less potent as an inhibitor of neutrophil elastase [33].

The Z-allele is a single mutation in exon 5, leading to substitution of the amino acid glutamine in position 342 in the protein for a lysine amino acid [11]. The S-allele is a single amino acid mutation in position 264 in the protein where a glutamine is replaced by a valine amino acid [30].

**Figure 6:**
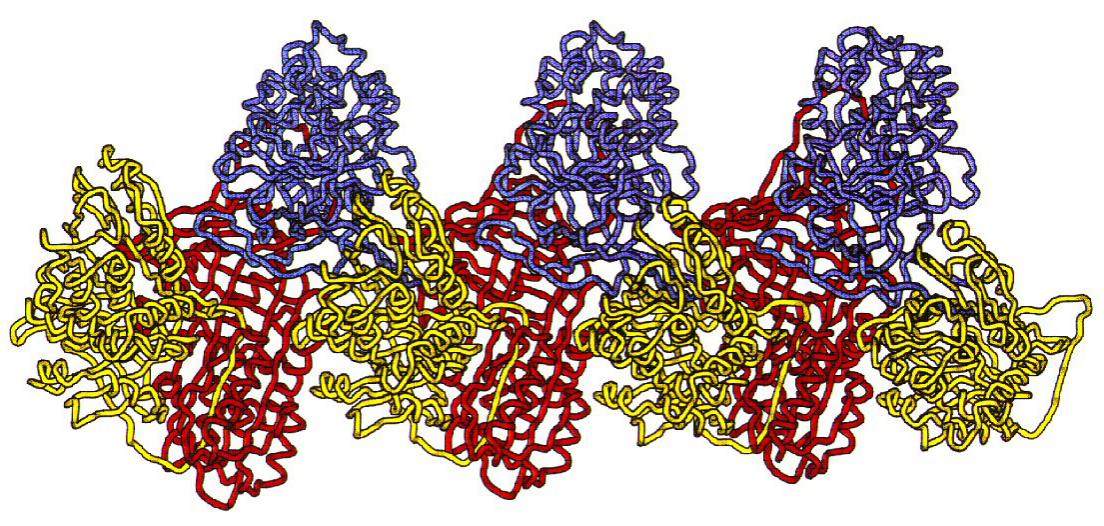
**Loop-sheet polymer of AAT molecules**. Reproduced from Lomas DA, Mahadeva R. Alpha-1-antitrypsin polymerization and the serpinopathies: pathobiology and prospects for therapy. *J Clin Invest*, 2002, 110:1585–90. Copyright ^© ^2002, with permission from American Society for Clinical Investigation.

What are the consequences of the S and Z allele? The homozygous S variant of alpha-1-antitrypsin results in an instable protein that is easily degraded outside the hepatocyte and affects the half-life of the S variant [30]. The homozygous Z variant has the property to form polymers to aggregate within hepatocytes and therefore this type of protein can only be secreted by hepatocytes in very low quantities [12] (Figure 2 and 6). Likewise, Z-type alpha-1-antitrypsin from monocytes also polymerize within these cells and can only be secreted at very low levels.

## Diagnosis and diagnostic methods

### Who should be tested how for alpha-1-antitrypsin deficiency?

**Figure 7:**
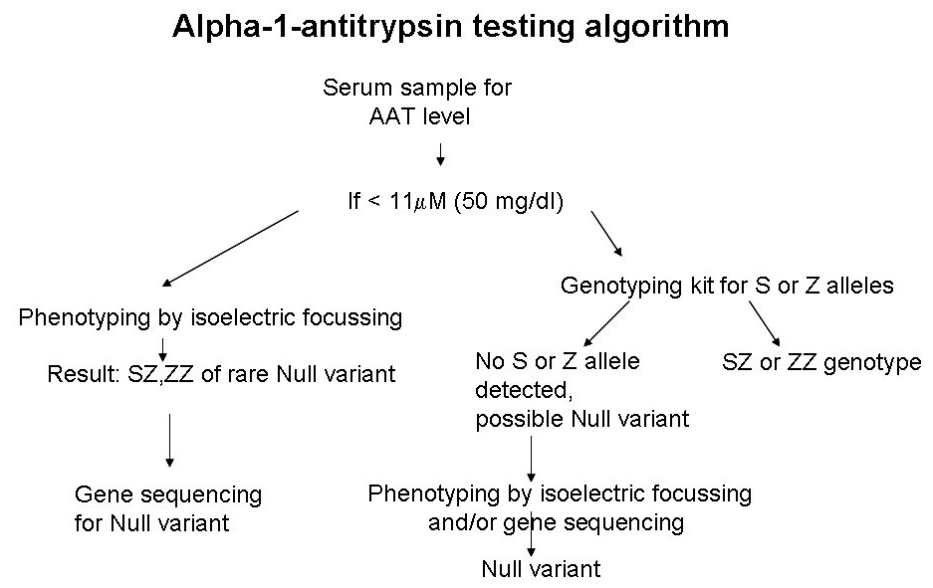
**Alpha-1-antitrypsin testing algorithm.** The first step is to measure the level in serum by radial immunodiffusion or nephelometry. If the serum level is below 11 **μ**M or 50 mg/ml (by nephelometry) further analysis by either isoelectric focussing or genetyping is warranted. Very low serum levels or levels below the detection limit of the serum assay suggest the presence of a Null variant (no production by the hepatocytes, and therefore distinct from the Z deficiency). A Null variant may be further analysed by isoelectric focussing and/or gene sequencing (as described in Fregonese *et al*., Respir Med; in press).

First of all, all newborns with a bleeding disorder or prolonged neonatal jaundice should be tested [10]. Secondly, all individuals with a history of asthma or chronic obstructive lung disease, particularly below the age of 40 [10]. Thirdly, all siblings of index cases [10]. Finally, all individuals with unexpected liver cirrhosis should be tested [2]. The aim of testing is to counsel individuals affected by the Z genotype. With respect to liver disease, patients should stop drinking alcohol and can be counseled about future liver transplant. With respect to lung disease, individuals should not start cigarette smoking or should stop urgently when smoking already started. This is the only available effective treatment to reduce the progression of decline in lung function. There is consensus in the scientific community on how to test, expressed in an ATS/ERS Statement on the diagnosis and treatment of alpha-1-antitrypsin deficiency [10]. This important document states that a physician should start with the detection of a serum level of alpha-1-antitrypsin (Figure 7). If reduced below the reference value of the laboratory, physicians should obtain a genotype or phenotype of alpha-1-antitrypsin. There are now commercial kits that test for presence of Z and S alleles in the gene [34]. If the serum level of alpha-1-antitrypsin is low, but the genotype test does not reveal a Z or S deficiency, isoelectric focusing of serum may reveal rare Null variants of alpha-1-antitrypsin which can be further characterized by genotyping (Figure 1).

Once new cases of alpha-1-antitrypsin have been detected, it is recommended by the Alpha1 International Registry (AIR) [35] to contact the national representative for alpha-1-antitrypsin deficiency matters to submit the clinical phenotype of the patient to the database of AIR and to obtain a plasma sample and DNA sample for future scientific studies and potential participation of the newly detected patient in future clinical trials for new drugs.

## Differential diagnosis

Often physicians diagnose patients with emphysema at age 30 to 40 but who have no alpha-1-antitrypsin deficiency. There are no known other genetic deficiencies that are as strongly related with emphysema as Pi ZZ alpha-1-antitrypsin deficiency. Pediatricians who see newborns with bleeding disorders or jaundice have a wider differential diagnosis. These include viral infection, hemochromatosis, Wilson's disease and autoimmune hepatitis. Rarely, liver biopsy is needed when Pi ZZ AATD is considered, because the latter can be easily determined by serum examination in the phenotyping laboratory.

## Genetic counseling and prenatal testing

When patients are identified as a new case of homozygous type Z alpha-1-antitrypsin deficiency, the issue of heritability for their children is frequently raised. It is less inconvenient for the children when first the other parent is investigated by isoelectric focusing or genotyping for alpha-1-antitrypsin. Since about 95% of individuals carry the MM phenotype, all children from parents with ZZ and MM type will carry the MZ type alpha-1-antitrypsin. If the parent is not MM, but is carrying a deficient allele next to the M allele (*i.e*. MZ), there is a 50% chance of ZZ genotype for every newborn from these parents and this can be confirmed in the child by isoelectic focusing of serum. Prenatal testing is not a routine procedure due to the low penetration of liver disease shortly after birth.

## Treatment

At present, treatment options for alpha-1-antitrypsin deficiency are very limited. There are no randomized, placebo-controlled studies that provide proof of an effective cure [10]. Only end-stage liver and lung disease can be treated by organ transplantation [10]. However, with the introduction of lung densitometry as a new outcome parameter for the evaluation of new drugs in lung disease, there are now, for the first time in 20 years, ongoing studies to investigate potentially efficacious new drugs in properly designed trials. We have to await the results of these trials before we can treat our patients effectively.

For treatment of lung disease, the ATS/ERS Statement recommends intravenous alpha-1-antitrypsin augmentation therapy for Pi ZZ individuals with FEV_1 _between 35 and 65% of predicted [10]. In addition, the World Health Organization (WHO) recommends annual flu vaccination and a pneumococcal vaccine every 5 years [3]. Like in emphysema patients without alpha-1-antitrypsin deficiency, relief of breathlessness may be obtained with long-acting bronchodilators and inhaled corticosteroids [36].

Patients with liver cirrhosis should be monitored for liver failure just like any form of liver cirrhosis and should be evaluated for liver transplant when they turn into clinical end-stage liver failure. Since the polymerization of Pi ZZ alpha-1-antitrypsin is accelerated by fever, patients may use paracetamol or other anti-inflammatory drugs to reduce fever.

## Natural history and prognosis

Alpha-1-antitrypsin deficiency with its many genotypes and its manifestation in various organs is rarely observed in daily clinical practice and is frequently not diagnosed or misdiagnosed. On average, the delay from the first signs of disease to the correct diagnosis is several years [37]. Once diagnosed correctly, the prognosis of both liver and lung disease is rather variable either. Several studies have shown that FEV1 is the most important predictor of survival of patients with emphysema due to alpha-1-antitrypsin deficiency (AATD). For individuals with an FEV1 below 20% of predicted, the 2 year mortality is 40% if not treated by a lung transplant [38]. Patients who have never smoked and who are detected by screening of affected family members turn out to have a normal life expectancy. Most of these AATD individuals (83%) are clinically healthy throughout adulthood and most will have liver enzyme abnormalities in early life [39]. All of these observations were performed more than 15 years ago and in the mean time computed tomography of the chest provided new analytical information on the quality of lung parenchyma, including the extent of emphysema. Dawkins *et al*. reported that lung density values assessed by computed tomography have better associations with mortality in type Z alpha-1-antitrypsin deficiency than FEV1 [40].

In adults, cirrhosis in AATD patients may become clinically apparent at any age, but the peak incidence is to be expected in the elderly age. Prognosis is generally grave, with a mean survival of 2 years after diagnosis of cirrhosis is established and is a clear indication for liver transplant [41].

## Competing interests

The authors declare that they have no competing interests.

## Authors' contributions

The two authors equally contributed to this review article. They read and approved the final version of the manuscript.
